# Biocompatibility of calcium phosphate bone cement with optimised mechanical properties: an in vivo study

**DOI:** 10.1007/s10856-016-5806-2

**Published:** 2016-11-14

**Authors:** Iwan Palmer, John Nelson, Wolfgang Schatton, Nicholas J. Dunne, Fraser Buchanan, Susan A. Clarke

**Affiliations:** 1 School of Mechanical and Aerospace Engineering, Ashby Building, Queen’s University of Belfast, 121 Stranmillis Road, Belfast, BT9 5AH UK; 2 Institute for Global Food Security, School of Biological Sciences, Medical Biology Centre, Queen’s University of Belfast, Lisburn Road, Belfast, BT9 7BL UK; 3 KliniPharm GmbH, Stephan Strasse 10, Frankfurt am Main, 60313 Germany; 4 School of Mechanical and Manufacturing Engineering, Stokes Building, Dublin City University, Collins Avenue, Dublin 9, Ireland; 5 School of Nursing and Midwifery, Medical Biology Centre, Queen’s University of Belfast, Lisburn Road, Belfast, BT9 7BL UK

## Abstract

This work establishes the in vivo performance of modified calcium phosphate bone cements for vertebroplasty of spinal fractures using a lapine model. A non-modified calcium phosphate bone cement and collagen-calcium phosphate bone cements composites with enhanced mechanical properties, utilising either bovine collagen or collagen from a marine sponge, were compared to a commercial poly(methyl methacrylate) cement. Conical cement samples (8 mm height × 4 mm base diameter) were press-fit into distal femoral condyle defects in New Zealand White rabbits and assessed after 5 and 10 weeks. Bone apposition and tartrate-resistant acid phosphatase activity around cements were assessed. All implants were well tolerated, but bone apposition was higher on calcium phosphate bone cements than on poly(methyl methacrylate) cement. Incorporation of collagen showed no evidence of inflammatory or immune reactions. Presence of positive tartrate-resistant acid phosphatase staining within cracks formed in calcium phosphate bone cements suggested active osteoclasts were present within the implants and were actively remodelling within the cements. Bone growth was also observed within these cracks. These findings confirm the biological advantages of calcium phosphate bone cements over poly(methyl methacrylate) and, coupled with previous work on enhancement of mechanical properties through collagen incorporation, suggest collagen-calcium phosphate bone cement composite may offer an alternative to calcium phosphate bone cements in applications where low setting times and higher mechanical stability are important.

## Introduction

Traumatic burst fractures of vertebral bodies accounts for approximately 15 % [1] of the estimated 19,000 annual spine and neck fractures which result in emergency admissions to hospital in England [2]. Such fractures are typically treated conservatively with a combination of analgesics, external bracing and bed rest; however, concerns regarding the inadequacies of these treatments have led to the increased indication of minimally invasive surgical procedures for these fractures [3].

Percutaneous vertebroplasty (PV), an image guided therapy involving the injection of bone cement into the vertebral body, is one such treatment. One indication for its use is osteoporotic compression fractures, where profound and rapid pain relief, coupled with an increase in functional activities, has been reported in up to 84 % of patients [4]. However, despite this, limited research has been conducted into the use of PV in the treatment of more severe traumatic burst fractures, consequently its routine application within the clinical setting is yet to be realised.

Currently, the cement of choice in PV is poly(methyl methacrylate) (PMMA), which displays good biocompatibility and haemocompatibility [5]. However, PMMA does have some significant drawbacks, including monomer toxicity, high polymerisation temperatures and an increased risk of fracture in adjacent vertebral bodies [6]. In addition, it is a relatively bioinert material, whereas bioactivity can be used to induce positive responses in vivo. This has led to the investigation of calcium phosphate bone cements (CPCs) for spinal and neck repair following burst fractures. CPCs mimic the mineral phase of bone and are resorbable [7], consequently promoting natural bone ingrowth and remodelling. As such they have the potential to be particularly effective in the treatment of traumatic burst fractures which, due to their typical mechanisms of injury, usually occur in the younger population who, in general, have a greater capacity for bone remodelling [8, 9]. However, despite this promise, concerns regarding limitations in their mechanical properties have limited the use of CPCs in the treatment of burst fractures [10–12].

Incorporation of collagen into CPCs is one way to enhance their mechanical properties. Approximately 95 % of the organic material in bone is type I collagen [13], which is involved in both bone function and formation. Collagen has been investigated as a biomaterial since the 1970s [14], both directly, in the form of hydrogels, and as an additive to other materials. Such work has shown that collagen, in part due to its low antigenicity and biocompatibility [15], has the potential to be highly beneficial in the field of bone regeneration.

It is hypothesised that, in addition to improving mechanical properties, collagen incorporation will give rise to biological benefits. Increased in vitro cellular adhesion has been observed as a result of bovine collagen incorporation into CPCs [16, 17]. Furthermore, an in vivo study has demonstrated a bovine collagen-CPC composite to be both biocompatible and resorbable [18].

Earlier work investigating both bovine collagen and collagen extracted from the marine Demosponge *Chondrosia reniformis* (Nardo, 1847 [19]) has shown that mechanical and handling properties of a novel CPC formulated from 100 % *α*-tricalcium phosphate (*α*-TCP-CPC) can meet several of the clinical requirements for PV through collagen incorporation (Table 1) [20–23], without compromising in vitro biological performance [24]. *C. reniformis* has been identified worldwide, has a high collagen content [25] and a low risk of detrimental toxic compounds [26]. The fact that this species also reproduces asexually suggests that harvesting of the sponge for collagen isolation could be conducted on a commercial scale [27]. Swatschek et al. [26] developed a method for isolating collagen from *C. reniformis* and demonstrated its suitability as a substitute for collagen from conventional sources. The collagen exhibits many features associated with mammalian collagen and, in some aspects, has been shown to display significant similarities to bovine collagen [28, 29]. Based on this and the earlier investigations, the aim of this study was to assess the in vivo biological response associated with collagen-*α*-TCP-CPC composites, formulated using either bovine or marine sponge collagens.

**Table 1:** Mechanical and handling properties of CPCs (modified from [21]) and typical values for PMMA bone cement (Colacryl B866, Lucite International Ltd., UK) [53, 54] (mean ± standard deviation)

Property	*α*-TCP-CPC	BC-CPC	MC-CPC	PMMA
Compressive strength (MPa)	31.6 ± 3.9	13.6 ± 2.3	37.8 ± 6.4	~60
Compressive modulus (MPa)	1145 ± 139	550 ± 51	1283 ± 211	>3000
Setting time (min)	29 ± 1.1	25.2 ± 0.5	15.5 ± 0.5	~15
Injectability (%)	52.6 ± 7.3	10.4 ± 1.5	55.9 ± 9.0	—

## Materials and methods

### Cement production

**Fig. 1:**
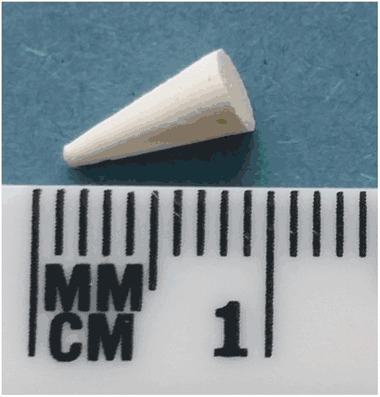
Conical cement sample (8 mm *high* with a base diameter of 4 mm) (color figure online)

*α*-TCP-CPC was produced following a previously described protocol [24] and shaped using polytetrafluoroethylene (PTFE) moulds. Conical cement samples, 8 mm high with a base diameter of 4 mm (Fig. 1), were produced for ease of implantation and because such geometry has been suggested for prediction of resorption by evaluating the short term resorption profiles [30]. A conical sample also has the advantage of more closely replicating the variable thickness of cement that would result from injection into vertebral cracks. Although such cements are injected minimally invasively and set in situ clinically, using pre-formed samples allowed precise control over sample geometry which facilitated analysis and comparisons.

Similar methods, also described by Palmer et al. [24], were used to produce the Collagen-CPC composites. Briefly, bovine derived collagen (Sigma-Aldrich, Gillingham, UK) or collagen extracted from *C. reniformis* (KliniPharm GmbH, Frankfurt, Germany) was incorporated into *α*-TCP-CPC at a loading of 1 wt% to form composites referred to as BC-CPC and MC-CPC respectively. Bovine collagen fibres were cryogenically ground using a Freezer Mill 6850 (Rondol Technology Ltd., Stoke-on-Trent, UK) for two cycles of 2 min, a method previously shown to reduce fibre length [31], prior to incorporation into the powder phase. Marine collagen fibres were suspended in the liquid phase of the cement before mixing with the powder phase.

A commercially available PMMA based cement routinely used clinically for PV, Vertebroplastic^®^ Radiopaque Resinous Material (DePuy International Ltd., Leeds, UK) (referred to herein as VP-PMMA), was used as a control. VP-PMMA was prepared using the same PTFE moulds, under aseptic conditions, as per the manufacturer’s instructions. All of the CPCs were sterilised using gamma (γ) irradiation in accordance with ISO 11137-1:2006 [32] (Isotron Applied Sterilisation Technologies, Synergy Health plc., Swindon, UK). This method of sterilisation has been reported as the most suitable for collagen extracted from *C. reniformis* [33].

### Model selection

New Zealand White rabbits were chosen as the in vivo lapine model (Thrush Hall Supplies, Co. Antrim, UK). All rabbits were skeletally mature females of approximately 28 weeks old [34], with a weight of 3.59 ± 0.06 kg (Mean ± Standard Error). All procedures had ethical approval and were conducted in accordance with the regulations defined in the UK Animals (Scientific Procedures) Act 1986.

### Surgery

A total of 38 rabbits underwent surgery. Two rabbits received implants unilaterally at the distal femoral condyle of the right leg. The remainder of the rabbits received bilateral implants. All defects were randomly assigned an implant type: *α*-TCP-CPC, BC-CPC, MC-CPC, VP-PMMA, or no implant, which was to confirm that the defects being used were critically sized. The rabbits were divided into two groups to be sacrificed 5 weeks and 10 weeks post-implantation. These two groups consisted of 18 and 20 rabbits respectively. The 5 week group were implanted with eight of each cement type and four empty defects; the 10 week group were implanted with eight of each cement type with six empty defects. These time points were selected as they have previously been shown to be appropriate for the assessment of new bone growth surrounding, as well as resorption of, CPCs in a lapine model [35].

**Fig. 2:**
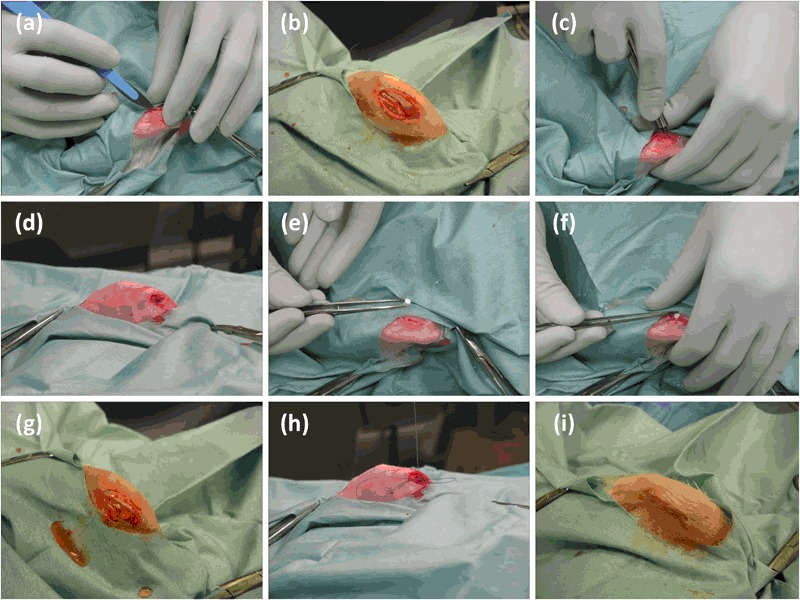
Surgical procedure. **a** and **b** Incision created medial to the patella. **c** and **d** Defect created by reaming with a hand-held drill. **e**, **f** and **g** Implant press fit into place until base of implant was flush with cortical bone. **h** Subcutaneous layer sutured with an interrupted pattern. **i** Skin sutured with a continuous subcuticular pattern (color figure online)

The procedure was similar to that used by Clarke et al. [36] and was conducted under aseptic conditions. Rabbits were sedated and anaesthetised using a neuroleptanalgesic, Hypnorm^®^ (VetaPharma Ltd., Leeds, UK), administered intramuscularly at 0.25 mL/kg and midazolam (HYPNOVEL^®^, Roche Products Ltd., Welwyn Garden City, UK), which was administered intravenously at 0.5–1.5 mg/kg. Both hind limbs were prepared for surgery by shaving, draping and swabbing the surgical site with a chlorhexidine based antimicrobial agent (ChloraPrep^®^, CareFusion UK 306 Ltd., Basingstoke, UK). An incision (~3cm) was made medial to the patella, allowing lateral dislocation of the patella. The femoral condyle was visualised and a defect matching the size of the implant created by reaming with a custom made hand-held steel drill. Prior to implantation the defect was lavaged with a saline solution to remove any bone debris, bone marrow and/or blood from the cavity. The implant was then inserted into the defect so that the base of the cone was flush with the cortical bone before the patella was reduced. The incision was closed in two layers; the subcutaneous layer was sutured with an interrupted pattern using absorbable sutures (Coated VICRYL™, Ethicon Inc., Livingston, UK); the same sutures were used to close the skin using a continuous subcuticular pattern (Fig. 2).

Following the procedure the rabbits were given a broad spectrum antibiotic (Baytril^®^, Bayer HealthCare AG, Leverkusen, Germany) at 2 mL/kg subcutaneously and an analgesic (Temgesic^®^, Reckitt and Coleman, Hull, UK) subcutaneously at 0.5 mL/kg mixed 1:9 with saline solution. Animals were monitored regularly postoperatively and returned to single housing cages, were weight bearing within approximately 2–3 h, and had access to food and water *ad libitum*.

### Sacrifice and sample collection

Animals were euthanised through the use of sodium pentobarbital (Euthatal, Merial Animal Health Ltd., Woking, UK), which was administered intravenously until permanent cessation of circulation, generally ~0.7 mL/kg. The implanted limbs were removed and cleaned of soft tissue. Femora were fixed in 4 wt% paraformaldehyde in phosphate buffered saline (PBS) (both Sigma-Aldrich, Gillingham, UK) for two days at 4 °C then stored in 70 % ethanol, also at 4 °C, until further examination.

### Histological analysis

Following fixation specimens were dehydrated through graded alcohols followed by acetone (Sigma-Aldrich, Gillingham, UK) before being embedded in a PMMA based resin (Technovit^®^ 9100 NEU, Heraeus Kulzer, Wehrheim, Germany) according to the manufacturer’s instructions. Embedded specimens were sectioned using an Accutom-50 precision cut-off machine (Struers Ltd., Rotherham, UK). Sections of 300 µm were cut longitudinally through each implant before being adhered to fully frosted microscope slides (Fisherfinest, Fisher Scientific Ltd., Loughborough, UK). Sections were then polished with an Alpha 2 Speed Grinder/Polisher fitted with a Vector™ Power Head (both Buehler, Coventry, UK) prior to staining.

Sections were stained with a 0.25 % w/v Toluidine Blue (TolBlue) solution (Sigma-Aldrich, Gillingham, UK) then rinsed with water and allowed to air dry before viewing. Enzyme histochemistry for tartrate-resistant acid phosphatase (TRAP), a commonly used technique for the detection of osteoclasts, was also performed. Prior to staining, resin was removed from the sections by soaking in acetone. Sections were adhered to fresh slides and rehydrated through graded alcohols to distilled water. The staining solution was prepared from a kit (Acid Phosphatase, Leukocyte, Sigma-Aldrich, Gillingham, UK) according to the manufacturer’s instructions. Sections were incubated in the reaction mixture at ambient temperature for 10–15 min until red stained areas were visible. Sections were then covered in an aqueous based permanent mounting medium (CC/Mount™, Sigma-Aldrich, Gillingham, UK) and dried at 70 °C.

TolBlue and TRAP stained sections were viewed under a Nikon SMZ800 stereomicroscope (Nikon UK Ltd., Kingston upon Thames, UK). Bone affinity index (AI) was calculated by measuring the percentage of the implant perimeter that was in direct contact with bone [37] using ImageJ (V. 1.46r, National Institutes of Health, USA). Analysis of the TRAP stained sections was conducted using Photoshop Elements 2.0 (Adobe Systems Europe Ltd., Maidenhead, UK). The implant and an area of surrounding bone up to 1.5 mm distance from the implant in each direction was selected and, within this selection, red pixels, which were indicative of TRAP activity, were quantified.

### Statistical analysis

One-way analysis of variance (ANOVA) was used to identify significant differences between cement formulations. Where a significant difference was indicated, pair-wise comparisons to assess inter-group differences were carried out using Tukey’s Honestly Significant Difference (HSD) tests provided the sample sizes were the same. If sample sizes were not consistent pair-wise comparisons were carried out using Gabriel’s procedure. All statistical analyses herein were performed using PASW Statistics 18.0 (IBM Corporation, New York, USA). A probability value of ≤0.05 was considered significant throughout.

## Results

### Histology

#### General observations

**Fig. 3:**
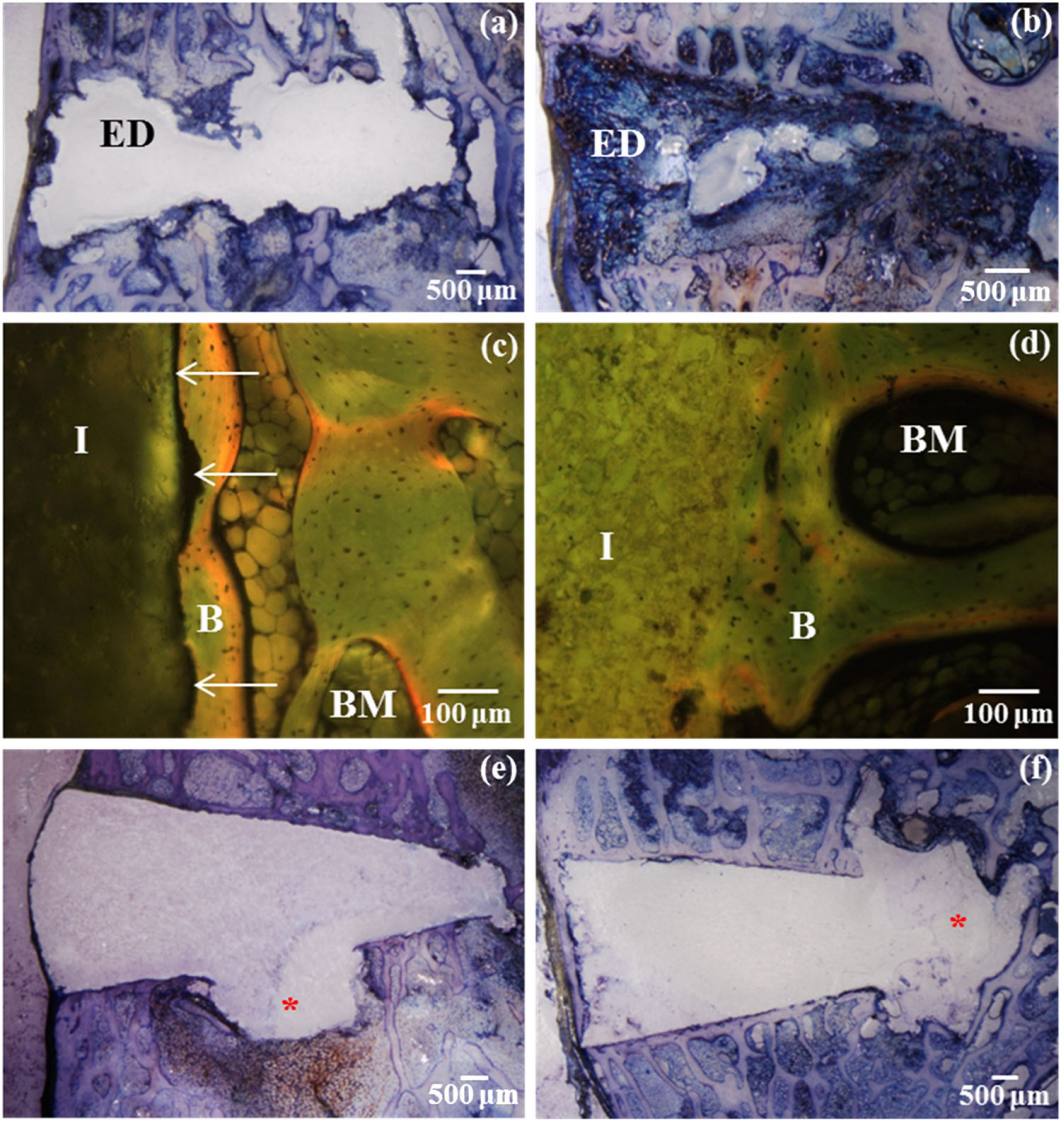
**a** and **b** Empty defects at the 5 and 10 week time points respectively showing little evidence of bone ingrowth. **c** Bone growth close, but not apposed, onto a VP-PMMA implant following 5 weeks implantation. *Arrows* indicate space between implant and bone. **d** Bone directly apposed onto a CPC based implant (BC-CPC) following 5 weeks implantation. **e** and **f** Voids observed around VP-PMMA implants after both 5 and 10 weeks implantation, respectively. *Asterisks* indicate voids. Key: *ED* empty defect, *B* bone, *BM* bone marrow and *I* implant (color figure online)

Histological analysis of the empty defects confirmed that the defects created were critically sized with little evidence of bone growth observed at either time point, excepting the cortical bone which had “closed” the top of the defect (Fig. 3a and b). At low magnifications there appeared to be similar bone apposition on each cement type. However, under higher magnification, in general, bone growth was occurring near the VP-PMMA implant but was not apposed onto the surface (Fig. 3c). Conversely, bone apposition onto the surfaces of the CPC implants was clearly visible (Fig. 3d).

Some evidence of an inflammatory response in the form of fibrosis around the implant, was observed, but was not confined to a particular implant type or time point. However, a phenomenon was observed in several sections from the VP-PMMA group at both 5 week and 10 week time points where the implants appeared to be associated with voids in the adjacent bone (Fig.3e and 3). 80 % of the 5 week sections showed such voids, decreasing to two-thirds at 10 weeks. Generally, but not exclusively, these voids were observed around the tips of the implants. Such voids were not observed around the CPC implants or the empty defects.

**Fig. 4:**
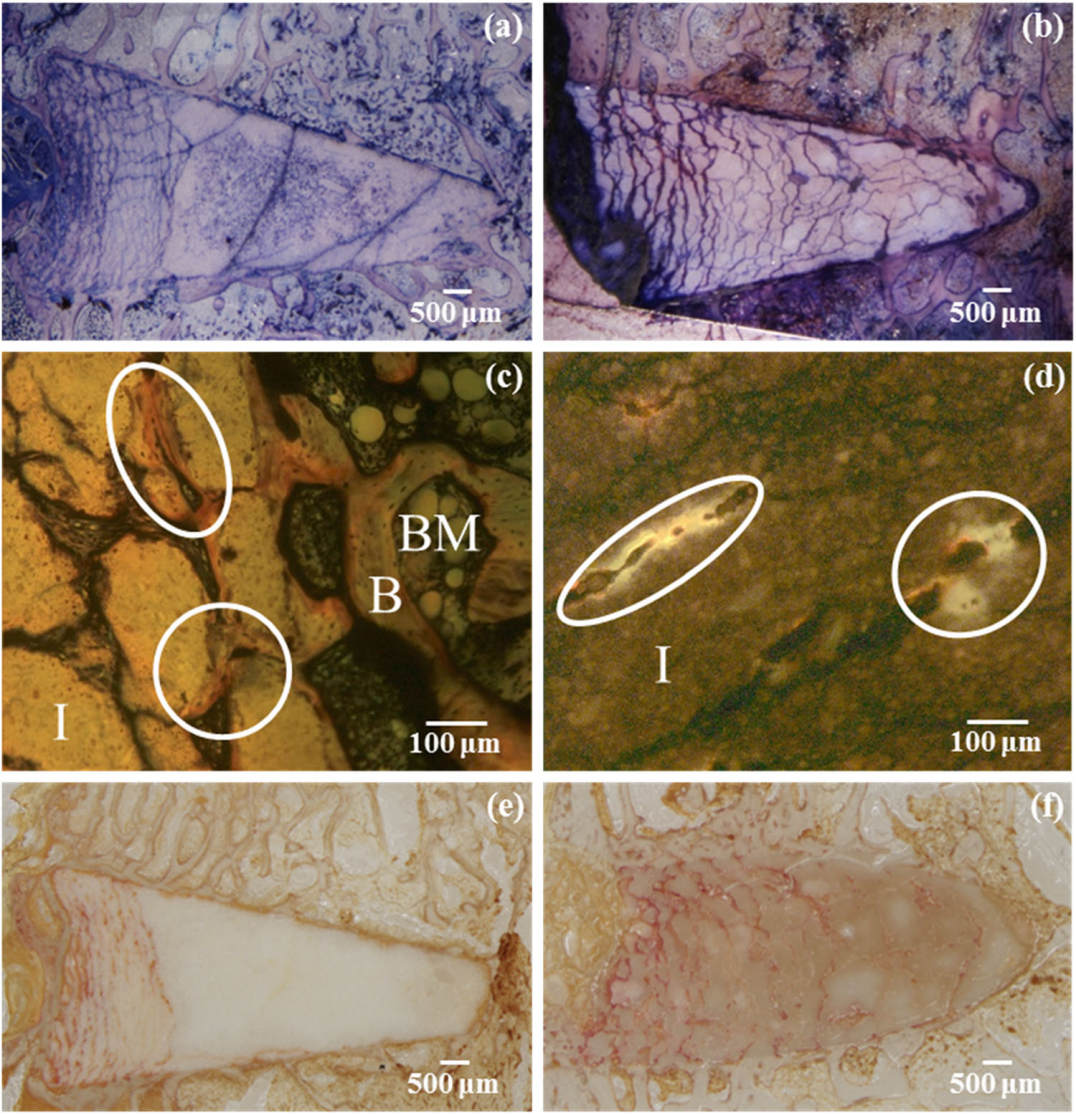
**a** and **b** Cracking of implanted CPCs after both 5 and 10 weeks implantation respectively. **c** Bone growth in cracks near implant edge. **d** Bone growth deeper inside implant. **e** and **f** *Red* stained TRAP positive cells within cracked CPCs after 5 and 10 weeks implantation respectively. Key: *B* bone, *BM* bone marrow, *I* implant (color figure online)

Although no voids were observed around the CPC implants, these implants did display a different phenomenon which was not present in the vast majority of VP-PMMA group. The majority of the CPC implants at both time points displayed differing degrees of crack formation within the bulk material. The extent of cracking ranged from very limited, to extensive cracking. A small number of implants displayed cracks to such an extent that the implant was broken into two fragments. In general, the majority of the cracks formed at the widest region of the implant nearest the outer cortical bone (Fig. 4a and b).

Where more extensive crack formation occurred some instances of bone growth into these cracks was noted. This generally occurred in cracks near the edge of the implant (Fig. 4c), but was also observed deeper within implants (Fig. 4d). In addition, TRAP activity was also observed in the cracks (Fig. 4e and f).

#### Histomorphometry

**Fig. 5:**
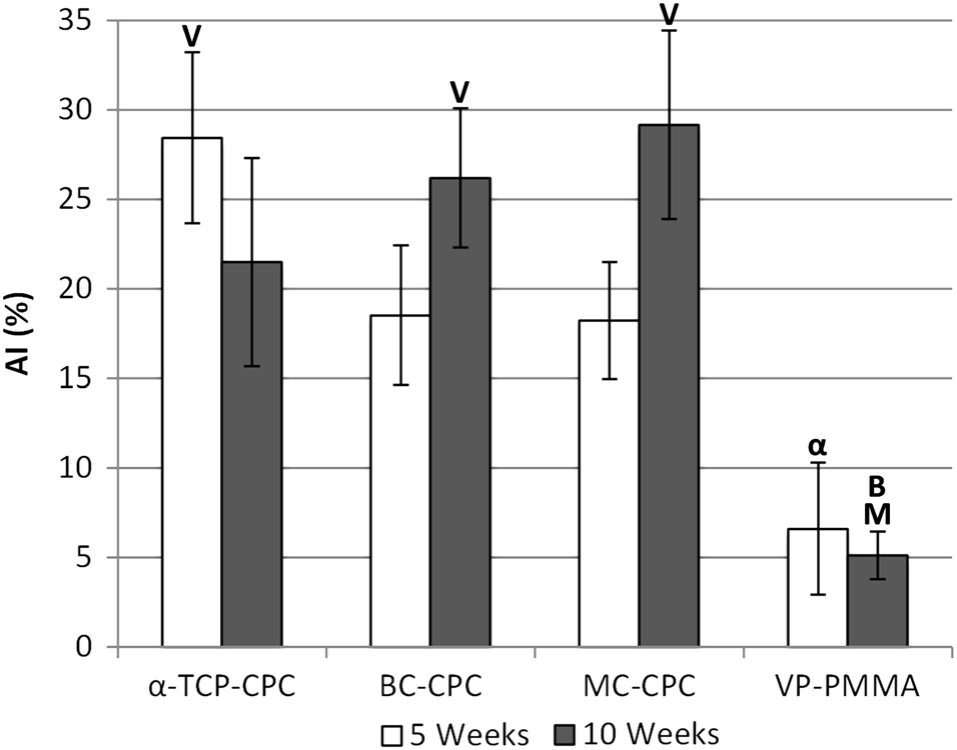
Bone affinity index (AI) of each cement type after 5 and 10 weeks implantation (mean ± standard error; *α*-TCP-CPC after 5 and 10 weeks, *n* = 6 and *n* = 7; BC-CPC after 5 and 10 weeks, *n* = 8 and *n* = 7; MC-CPC after 5 and 10 weeks *n* = 6 for each; VP-PMMA after 5 and 10 weeks, *n* = 5 and *n* = 6). V denotes a significant difference between the labelled bar and VP-PMMA at the same time point; *α* denotes a significant difference between the labelled bar and *α*-TCP-CPC at the same time point; B denotes a significant difference between the labelled bar and BC-CPC at the same time point; M denotes a significant difference between the labelled bar and MC-CPC at the same time point

In general, AI was higher for the CPC formulations when compared with the VP-PMMA group. Following 5 weeks implantation the mean AI was approximately 180 % higher in both collagen augmented CPCs than in the VP-PMMA group; this increased to around 330 % in the *α*-TCP-CPC group. Following 10 weeks implantation the mean AI values for the *α*-TCP-CPC, BC-CPC and MC-CPC groups were approximately 320, 410 and 470 % higher, respectively, than that of the VP-PMMA group. Despite this, relatively large variations within groups resulted in few statistically significant differences. Following 5 weeks implantation no significant difference in AI was observed between the three CPC formulations. Similarly, no significant difference in AI was observed when VP-PMMA was compared against both BC-CPC and MC-CPC. However, *α*-TCP-CPC displayed a mean AI significantly higher than VP-PMMA (*P* = 0.01). Following 10 weeks implantation there remained no significant difference between the AI values of the CPC formulations. However, both BC-CPC and MC-CPC demonstrated AI values significantly higher than VP-PMMA (*P* = 0.007 and *P* = 0.01 respectively). In addition, for each implant type, no significant increase in AI was observed between 5 and 10 weeks implantation (Fig. 5).

**Fig. 6:**
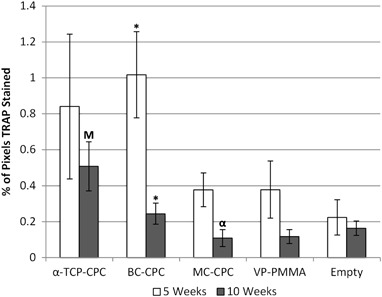
Percentage of pixels TRAP stained within 1.5 mm of the implant (mean ± standard error; *α*-TCP-CPC after 5 and 10 weeks, *n* = 6; BC-CPC after 5 and 10 weeks *n* = 7; MC-CPC after 5 and 10 weeks, *n* = 5 and *n* = 4; VP-PMMA after 5 and 10 weeks, *n* = 5; Empty defect after 5 and 10 weeks, *n* = 3 and *n* = 5). *α* denotes a significant difference between the labelled bar and *α*-TCP-CPC at the same time point; M denotes a significant difference between the labelled bar and MC-CPC at the same time point; * denotes a significant difference between time points within a sample group

TRAP activity was reduced between 5 and 10 weeks (Fig. 6); however, as a result of the large variations, the only statistically significant decrease between time points was observed in the BC-CPC group (*P* = 0.019). TRAP activity in the MC-CPC, VP-PMMA and empty defect groups were similar with a trend towards higher activity in both *α*-TCP-CPC and BC-CPC groups.

## Discussion

The aim of this study was to assess the in vivo performance of CPCs with enhanced mechanical properties through the incorporation of collagen. In general, AI was higher for the CPC based formulations than in the VP-PMMA group. When the VP-PMMA implants were investigated bone growth was observed in close proximity to the implant (generally within 100 μm) but was rarely apposed onto the implant. This finding is likely to be due to the good in vivo osteoconductivity of CPCs [7, 38–42] coupled with the lack of osteoconductivity associated with PMMA bone cement [43]. These findings are in agreement with the work of Tsai et al. [35], who noted excellent bonding between a CPC and host bone in a rabbit model after 12 weeks implantation. Similarly, in a study involving the injection of a CPC into a canine model, the surface of the cement was almost entirely covered with bone within 14 days of implantation [44].

The results suggest that incorporation of collagen may initially slow bone apposition compared to the native cement, but, by 10 weeks, apposition onto both collagen-CPC composites matched that of the native CPC. This finding is in line with the results of previous in vitro work showing that cells were able to proliferate equally well on all of the CPCs as well as differentiating on both *α*-TCP-CPC and MC-CPC, albeit at shorter time points [24].

Few studies have assessed the incorporation of collagen into CPCs. Mai et al. [18] incorporated bovine collagen into Calcibon resulting in good bone integration, but the absence of an non-augmented control meant that the effect of collagen incorporation could not be quantified. In vitro analyses into the effect of collagen incorporation into CPCs have generally focused on handling and mechanical properties [16, 17, 20, 45]. However, it has been shown that incorporation of bovine collagen into CPCs can improve cellular adhesion capacity [16, 17]. To the best of the authors’ knowledge, this is first in vivo study investigating the incorporation of collagen from *C. reniformis* into a CPC. As such the effect of this collagen on the in vivo behaviour of MC-CPC is based solely on the experimental results presented herein.

TRAP staining results suggest that the CPCs were not being actively resorbed. This is not surprising given that the CPCs set to form calcium deficient hydroxyapatite (CDHA), which resorbs very slowly [46]. However, unlike PMMA, CDHA is eventually remodelled, a process that may be accelerated given that PV results in thin tendrils of cement injected into cracks within a vertebral body rather than a bulk implant.

A general trend towards decreased TRAP activity over time was observed. This may be a result of the waning of initial trabecular bone repair. The trend towards a declining osteoclast response over time suggests that active resorption of the CPCs would indeed be a long-term remodelling process. However, it must be considered that a relatively consistent level of activity represents normal cellular function around an orthopaedic injury; a suggestion which is reinforced by the TRAP activity observed round the empty defects. Despite this, it should be noted that TRAP activity is not confined to osteoclasts [47, 48]; TRAP expression is associated with the differentiation and activation of cells of monohistiocytic phenotype which includes macrophages, among others, in addition to osteoclasts. It is thus possible that some of the TRAP activity observed may be as a result of macrophages initiating the inflammatory response associated with the injury. However, given that the first time point considered was 5 weeks, it is likely that any initial inflammatory response would have subsided by this point. Also, qualitatively, only limited signs of such a response were observed when the sections from both time points in this study were examined. Immunohistologic examination of the sections would be required to confirm or refute this postulation.

In general, the CPCs investigated herein showed extensive bone apposition, suggesting that these materials are well accepted in vivo. Although no clear evidence of fibrous encapsulation was observed in the VP-PMMA group, the differences observed in bone bonding between these materials and the CPCs may be an indication that they were not as well tolerated in vivo. In addition to the lack of bone apposition onto VP-PMMA implants, this material appeared to result in the formation of voids adjacent to the implants. The cause of these voids remains unclear, but it is possible that residual unreacted monomer was leaching out of the implants and resulting in tissue necrosis. This is thought unlikely as VP-PMMA implants were produced in advance of the operative procedures, which would allow time for the evaporation of any residual monomer which may have been present; no histological evidence of necrosis was observed either. The voids are also unlikely to be artefacts from section preparation due their exclusive association with VP-PMMA implants. Although the exact cause of such voids has not been confirmed, the lack of voids in the CPC implants suggests that they are better tolerated in vivo than the VP-PMMA implants.

The occurrence of cracks was far more prevalent in the CPC implants, with only minimal cracks observed in the VP-PMMA group; this was expected due to differences in mechanical properties. Concerns have been raised regarding the mechanical properties, particularly the fracture toughness of CPCs, however, the non-augmented formulation used herein has been shown to be within the clinical range required for vertebroplasty [49]. Furthermore, incorporation of both bovine collagen and collagen from *C. reniformis* has been shown to improve the mechanical properties of this CPC [20, 50]; although, in terms of the crack formation, no clear differences were observed in this study. Given that the CPCs investigated have demonstrated in vitro mechanical properties within the clinical range required for PV, the crack formation observed is not considered a concern. Cracks forming within the implant may in fact be a positive outcome by allowing cellular infiltration into the implant. The majority of the cracks being located near the base of the implant are likely a result of cortical bone growth around the top of the implants, resulting in more stress on this part of the implant. Where extensive cracking was observed, some instances of TRAP staining and bone growth were noted within the cracks. A loss of mechanical loading in bone can be sensed by osteocytes, which play a key role in bone remodelling [51, 52]. It is possible that the formation of cracks within the implants resulted in perturbations in mechanical loading in the apposed bone causing the onset of remodelling, as osteocytes have been suggested to determine which surfaces are resorbed by osteoclasts [52]. Movement of osteoclasts to such areas near the implant may lead to subsequent migration into the cracks with many of the cracked implants showing TRAP positive cells in these regions. This would suggest that osteoclasts are present within the cracks and are attempting to resorb areas of the implant. Localised regions of bone growth, which were also observed within the cracks, indicate that the osteoclast activity is being followed by the deposition of new bone by osteoblasts.

## Conclusions

In general, all implant types were well tolerated in vivo with little inflammatory response observed. However, the majority of VP-PMMA implants at 5 week and 10 week time points were associated with voids in the adjacent bone. No such voids were associated with the CPC formulations suggesting that they are better tolerated. Crack formation to a varying degree was observed in the majority of implants, though extensive cracks within the implant were mainly confined to the CPC formulations. This behaviour was not considered to be a concern and may in fact prove beneficial by allowing cellular infiltration into the implant. Bone apposition onto the implants confirmed that the CPCs were well tolerated in vivo and this was not compromised by the incorporation of collagen from either source.

These findings, coupled with the knowledge that addition of marine collagen significantly improves the mechanical properties of *α*-TCP-CPC, suggests that it may be a suitable alternative to some of the most popular commercially available bone cements in applications such as PV, where mechanical stability is essential.
